# The anti-inflammatory effect of kaempferol on early atherosclerosis in high cholesterol fed rabbits

**DOI:** 10.1186/1476-511X-12-115

**Published:** 2013-07-29

**Authors:** Lingxi Kong, Cheng Luo, Xiuying Li, Yuanda Zhou, Haixia He

**Affiliations:** 1 Clinical Pharmacology Laboratory, Department of Pharmacy, The First Affiliated Hospital of Chongqing Medical University, Chongqing, PR China

**Keywords:** Kaempferol, Atherosclerosis, Inflammation, Vascular adhesion molecule

## Abstract

**Background:**

Atherosclerosis has been widely accepted as an inflammatory disease of vascular, adhesion molecules play an important role in the early progression of it. The aim of the present study was to evaluate the effect of kaempferol on the inflammatory molecules such as E-selectin (E-sel), intercellular adhesion molecule-1 (ICAM-1), vascular cell adhesionmolecule-1 (VCAM-1) and monocyte chemotactic protein-1 (MCP-1) in high cholesterol induced atherosclerosis rabbit models.

**Methods:**

Thirty male New Zealand white (NZW) rabbits were randomly divided into five groups, control group, model group, fenofibrate (12mg/kg) group and kaempferol groups (150 mg/kg and 30 mg/kg). The rabbits were fed with a normal diet or a high cholesterol diet for 10 weeks. Levels of blood lipids, serum tumour-necrosis factor-alpha (TNF-α) and serum interleukin-1beta (IL-1β) were detected at the end of the sixth and tenth week. Malonaldehyde (MDA) level and superoxide dismutase (SOD) activity in serum were also determined. Lesion areas of the aorta were measured with morphometry analysis after ten weeks. Gene expression of E-sel, ICAM-1, VCAM-1 and MCP-1 in aortas was determined by RT-PCR (reverse transcription-polymerase chain reaction). Immunohistochemical staining was employed to measure protein expression of E-sel, ICAM-1, VCAM-1 and MCP-1.

**Results:**

Model rabbits fed with ten weeks of high-cholesterol diet developed significant progression of atherosclerosis. Compared with the control, levels of blood lipids, TNF-α, IL-1β and MDA increased markedly in serum of model rabbits, while SOD levels decreased. Gene and protein expressions of E-sel, ICAM-1, VCAM-1 and MCP-1 in atherosclerotic aortas increased remarkably in model group. However, comparing to the model rabbits, levels of TNF-α, IL-1β and MDA decreased significantly and serum SOD activity increased, gene and protein expressions of E-sel, ICAM-1, VCAM-1 and MCP-1 in aortas decreased significantly with the treatment of kaempferol.

**Conclusion:**

Kaempferol shows anti-atherosclerotic effect by modulating the gene and protein expression of inflammatory molecules.

## Background

Cardiovascular disease (CVD) continues to be the leading cause of death in developed countries nowadays. As the most important contributor of CVD, atherosclerosis is arousing great attention worldwide. With the development and progression of atherosclerosis, a great deal of lipid deposits and fibrous plaques accumulate in arteries of some critical organs, especially heart and brain, leading to the formation of thrombus, which is responsible for most of the breakouts of clinical vascular events [1].

Atherosclerosis is currently well accepted as a chronic inflammation of arteries. The lesions of atherosclerosis represent a protective and inflammatory response against different and multiple risk agents including cholesterol, elevated oxidated LDL, free radicals caused by cigarette smoking, hypertension, diabetes and infections [2]. A recent report found some new risk factors in the pathogenesis of atherosclerosis such as homocysteinemia, elevated plasma levels of lipoprotein (a) [Lp(a)], fibrinogen, impaired fibrinolysis, increased platelet reactivity and hypercoagulability [3].

Inflammatory mechanisms play a critical role in the pathogenesis of atherosclerosis. In the early phase of atherosclerosis, dysfunction of endothelium leads to the increased permeability of endothelium and adhesiveness of leukocytes to artic wall. The production of procoagulant agents including vasoactive molecules, cytokines and growth factors can also be induced in endothelium. Blood monocytes adhere to epithelium, migrate into intima and become intimal macrophages, then change to foam cells by excessive ingestion of modified lipoprotein and gradually form the so called fatty streak [2].

The initial stage of inflammation of atherosclerosis is usually silent and long with increased adhesion of monocytes in arterial endothelium [4]. Adhesion molecules and monocyte chemotactic protein-1 (MCP-1) participate in the progress and play an important role. Fisrtly, selectin including E-selectin (E-sel), P-selectin(P-sel), and L-selectin (L-sel) facilitate the rolling of molecules on the surface of endothelium cells, then adhesion molecules, such as vascular adhesion molecule-1 (VCAM-1) and intercellular adhesion molecule-1 (ICAM-1) mediate the advanced and firm adhesion of monocytes. Lastly, with the function of MCP-1, monocytes pass though endothelium and migrate into intima [5,6]. Damaged endothelial cells, arterial lesions from atherosclerosis experiment model and human all show increased expression of these molecules including E-sel, ICAM-1, VCAM-1 and MCP-1 [7,8]. Therefore, one possible mechanism for ameliorating atherosclerosis is the down-regulation of pro-atherogenic molecules such as E-sel, ICAM-1, VCAM-1 and MCP-1.Besides, in the inflammatory progress of atherosclerosis, activation of endothelial cells and mactophages leads to the release of various kinds of cytokines, chemokines, and growth factors, which in return regulate the continued and advanced accumulation and migration of leukocytes, thus induce further inflammation [2]. Pro-inflammatory cytokines such as tumour necrosis factor-alpha (TNF-α) and interleukin-1beta (IL-1β) participate in it by inducing E-sel, ICAM-1 and VCAM-1 expression in endothelial cells [9], releasing of other inflammatory mediators and inducing apoptosis of endothelial cells. Previous studies revealed that elevated levels of TNF-α and IL-1β in patients with CVD have been detected [10,11], indicating that TNF-α and IL-1β are two important cytokines in vascular inflammation and highly associated with atherosclerosis. Recent studies also suggested that, inflammatory biomarkers such as TNF-α, IL-1β, E-sel, ICAM-1, VCAM-1 and MCP-1 may play a potential role for the prediction of risk for developing CVD and may correlate with the severity of CVD [12,13].

Kaempferol(3,5,7-trihydroxy-2-(4-hydroxyphenyl)-4H-1-benzopyran-4-one), a yellow compound with a low molecular weight (MW: 286.2 g/mol), is a common natural flavonoid. This flavonoid is abundant in many plant-derived foods and traditional medicine. It has been reported that kaempferol and its glycosides have many kinds of pharmacological activities, such as antioxidant, anti-inflammatory, anticancer, antimicrobial, neuroprotective, antidiabetic, analgesic and anti-allergic activities [14]. And results of some studies suggested that the dietary intake of kaempferol-rich foods can reduce risk of developing several disorders including cardiovascular diseases [15,16].

Therefore we considered kaempferol to be a potent and effective agent against atherosclerosis. So far, the researches about the antioxidant, anti-inflammatory and cardioprotective effect of kaempferol have been mostly centered on endothelium cells in vitro. However, in vivo studies related to the anti-atherosclerosis effect of kaempferol are so rare. So the aim of the present study was to investigate the protective effect of kaempferol on atherosclerosis rabbit models. And our study will be focused on the anti-inflammation effect and its influence on the expression of inflammatory molecules E-sel, ICAM-1, VCAM-1 and MCP-1 in the aorta of rabbits. Besides, Fenofibrate was designed as a positive control in our study.

As a commonly used and successful atherosclerosis animal model, New Zealand White rabbits were chosen in our experiment. The lipoprotein metabolism and cardiovascular system of rabbits are much similar to those of humans, and they have been proved as the excellent model for human atherosclerosis [17,18].

## Materials and methods

### Animals and experimental procedures

This study was approved by the Animal Ethics Committee, Chongqing Medical University. Thirty male New Zealand White rabbits with average body weight between 2.0± 0.5 kg were purchased from Experimental Animal Center, Chongqing Medical University (Grade II, Certificated SCXKYu 2012–0001). High-cholesterol diet (normal diet supplemented with 1% cholesterol, 10% yolk powder, 5% animal oil) was supplied by Experimental Animal Center, Chongqing Medical University. The animals were housed separately in cages under standard animal house conditions with a 12 h light /dark cycle at the temperature of 25°C. All animals had free access to water.

**Figure 1:**
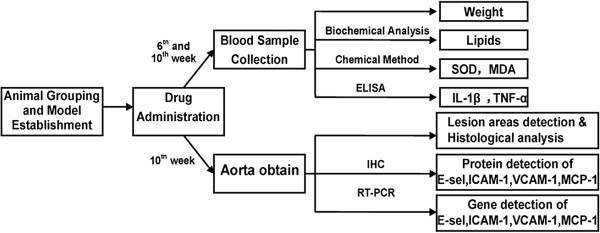
**Flow chart describing the experimental design.** ELISA: Enzyme-linked immunosorbent assay; RT-PCR: reverse transcription-polymerase chain reaction; IHC: Immuohistochemistry.

The rabbits were all fed on a normal diet for one week for adaptation, subsequently were divided randomly into five groups of six animals in each: control group (Control) was fed a normal diet, model group (Model) was fed with high cholesterol diet, fenofibrate group (Fenofibrate, positive control group) was fed with high-cholesterol diet plus fenofibrate (Laboratoires Fournier S.A., Dijon, France; 12 mg/kg/day), kaemlferol group of high dosage (Kaempferol-H) and kaempferol group of low dosage(Kaempferol-L) were fed with similar high-cholesterol diet plus different doses of Kaempferol (ZeLang Medicine Science and Technology Co., Ltd., Nanjing, China),150 and 30 mg/kg/day respectively. All the groups were fed with a diet of 120 g/day for ten weeks. The main experimental procedures were described in a flow chart as shown in Figure 1.

At the end of the tenth week, all animals were fasted overnight and sacrificed by intravenous injection of pentobarbital (Laboratories, Urchem Ltd., Shanghai,China, 60 mg/kg body weight).Immediately after opening the chest, the aorta was excised, then dissected longitudinally and separated into three portions. The first portion, a one-centimeter segment between the first and the second intercostals artery was fixed in 10% neutral buffered formalin for 1 day and paraffin-embedded; and it was used for histological and immunohistochemical analysis. The second portion, a segment between the second and the third intercostals artery, was snap-frozen in liquid nitrogen and stored at −70°C until being used for PCR. The third portion, a segment between the third and the seventh intercostals artery was stained with oil red O (sigma, USA) for 30 minutes, then transferred to 70% alcohol for differentiation. 15 min later, it was washed and taken photo with a digital camera (Olympas, Janpan). The percentage of oil red O-positive area to the internal surface was measured with a computer-assisted morphometry system (Image Pro Plus 6.0, USA).

### Weight and serum lipids determination

At the end of the sixth and tenth week, all the rabbits were weighted and 2.0 mL blood was taken via the marginal ear vein from each rabbit and centrifuged at 8,000 g for 10 min. Obtained serum was analyzed in an automatic blood chemical analyzer (Beckman Instruments, USA) and the serum concentrations of total cholesterol (TC), triglyceride (TG), high density lipoprotein cholesterol (HDL-C) and low density lipoprotein cholesterol (LDL-C) were determined.

### Evaluation of serum TNF-α and IL-1β

At the end of tenth week, TNF-α and IL-1β concentrations in the collected serum were detected with commercially available kits (Rapidbio Lab Co., California, USA) under the manufacturer’s instructions by the method of Enzyme-linked immunosorbent assay (ELISA). Values of samples were calculated from a standard curve generated from standards of known concentrations. Absorbance at 450 nm was detected using microplate reader (Bio-Rad, USA).

### Evaluation of serum SOD and MDA

After six and ten weeks, SOD and MDA levels in the collected serum of rabbits were detected by colorimetric method of xanthine oxidase and thiobarbituric acid with kits (Nanjingjiancheng Bioengineering Institute, Nanjing, China) respectively. The absorbance was measured with UV–vis spectrophotometer (Shimadzu,Japan) at the wavelength of 550 nm and 532 nm respectively, according to the instructions of the kits.

### Histological and immunohistochemical analysis

The first aorta portion, the paraffin-embedded segment between the first and the second intercostals artery, was cut into five non-consecutive serials of 5-μm-thick sections. The first serial was stained with hematoxylin and eosin for observation under light microscopy (Olympas, Japan). The other four serials of sections were incubated with primary antibodies to E-sel (Bioss, Beijing, China; 1:100 dilution), ICAM-1 (Bioss, Beijing, China; 1:100 dilution), VCAM-1 (Abcam Inc., Cambridge, USA; 1:100 dilution) or MCP-1 (Bioss, Beijing, China;1:100 dilution) respectively. After washing, specific biotinylated secondary antibodies (biotinylated goat anti-mouse IgG for VCAM-1, and biotinylated goat anti-rabbit IgG for E-sel, ICAM-1 and MCP-1) were applied, followed by avidin-biotin peroxidase complexes (Histostain™-Plus Kits, Beijing Zhongshan Goldenbridge Company, China). Antibody binding was visualized with 3, 3-diaminobenzidin (DAB). Sections were counterstained with hematoxylin. After scanned to computer, percentage of immunoreactive cells and staining intensity of each section were measured with use of a computerized image analysis program (Image-Pro Plus 6.0, USA).

For the immunohistochemical semiquantitative assessment of E-sel, ICAM-1, VCAM-1 and MCP-1expression, immunohistochemical score (HIS, ranging from 0–12) was applied. It is calculated by multiplication of the percentage of immunoreactive cells (quantity score) with the staining intensity (staining intensity score). Quantity score was calculated based on the following scoring system: no staining is scored as 0, 1–10% of cells stained scored as 1, 11–50% as 2, 51–80% as 3, and 81–100% as 4. Staining intensity is rated on a scale of 0 to 3, with 0 = negative; 1 = weak; 2 = moderate, and 3 = strong.

### Determination of E-sel, ICAM-1, VCAM-1 and MCP-1 gene expression

Total RNA was isolated from the second portion of aorta using TRIZOL (DingGuo Biotechnology Co. Ltd, Beijing, China). To avoid the interference of DNA, 4 μL of the obtained RNA was dealt with DNase (Promega, Madison, USA) in 10 μL reaction mixture before reverse transcription. Subsequently, 4 μL of the DNA-free RNA were reverse transcribed into cDNA in 20 μL reaction mixture using a reverse transcriptase (Toyobo, Osaka, Japan). Reverse transcription was carried out for 10 min at 30°C, 60 min at 42°C, followed by an inactivation step at 99°C for 5 min. Target gene expressions were determined by semi-quantitative PCR with β-actin as an internal standard. PCR amplification was performed on an Gene Cycler (Bio-Rad, USA) in a total volume of 25 μL,which was composed of 1.0 μL of cDNA template, 10 × PCR buffer (50 mM KCl, 10 mM Tris–HCl, 2.5 mMMgCl_2_, pH 8.3), 0.2 mM dNTPs (Genview scientific Inc., USA), 20 pmol/μL forward primers, 20 pmol/μL reverse primers,1.0 U of Taq DNA polymerase (TaKaRa Co. Ltd., Tokyo, Japan). The amplification procedure consisted of an initial denaturation step for 2 min at 94°C, 35 cycles of denaturation at 94°C for 30 sec, annealing at the temperature indicated in Table 1 for 1 min, and extension at 72°C for 30 sec, followed by a final extension step for 10 min at 72°C. Electrophoresis was carried out at 5 V/cm for 30 min on a 2% agarose gel and PCR products were visualized with silver staining. Absorbances of each band were determined by densitometric analysis using the one-Dscan gel analysis software (Scanalytics, Billerica, USA). mRNA levels were expressed as the ratios between target genes and β-actin.

**Table 1:** Primers sequence of target genes

**Target genes**	**Gene bank accession no.**	**Primers**	**Annealing temperature (°C)**	**Size (bp)**
β-Actin	NM_001101683.1	5′ -TTCCAGCCCTCCTTCCT- 3′	56	315
		5′ -GCCCGACTCGTCATACT- 3′		
E-sel	NM_001082312.1	5′ - AATGGCAGATACAGAGAACT- 3′	49	219
		5′ -TGGCTTGGAAGAGAATAACT- 3′		
ICAM-1	AB128157.1	5′ -GACATTCTTGAACAGTGACAG- 3′	46	183
		5′ -CGGACACAGCTCTCAGTA- 3′		
VCAM-1	NM_001082152.1	5′ -GGAGACACTGTCATTATCTCCTG- 3′	58	336
		5′ -TCCTTTCATGTTGGCTTTTCTTGC- 3′		
MCP-1	M28883.1	5′ -GGTGTAAAGGCAGGTGTG- 3′	52	214
		5′ -AGGATAGGAAAGGATGGG- 3′		

### Statistical analysis

Results were expressed as mean±SD. Statistic analysis was carried out using the SPSS statistical package version 10.0 (SPSS Inc., USA). Student’s t-test was performed to compare means between two groups. The p value<0.05 or 0.01 was considered to be statistically significant.

## Results

### Body weight and serum lipids analysis

Bodyweight and serum lipids were measured after six and ten weeks respectively, and the obtained data were shown in Table 2. According to Table 2, there was no significant difference in bodyweight among any of the groups at t = 6 week and at t = 10 week (p > 0.05). After feeding on high cholesterol diet for 6 and 10 weeks, serum lipid levels including total cholesterol (TC), triglyceride (TG), high density lipoprotein cholesterol (HDL-C) and low density lipoprotein cholesterol (LDL-C) of model rabbits all increased greatly (P<0.01) compared with control rabbits. Treatment of fenofibrate (12 mg/kg) or kaempferol (30 mg/kg and 150 mg/kg) for six and ten weeks significantly lowered TC, TG, HDL-C and LDL-C levels of rabbits in comparison to the model group (p < 0.01 or P<0.05).

**Table 2:** Changes of body weight and serum lipid levels after six and ten weeks in different groups (mean± SD, n=6)

	**Control**	**Model**	**Fenofibrate**	**Kaempferol-H**	**Kaempferol-L**
Bodyweight (kg)					
Week 6	2.49 ± 0.22	2.82 ± 0.36	2.65 ± 0.24	2.68 ± 0.28	2.66 ± 0.25
Week 10	2.93 ± 0.15	3.24 ± 0.40	3.15 ± 0.33	3.10 ± 0.13	3.09 ± 0.24
TC (mmol/L)					
Week 6	1.13 ± 0.33	6.01 ± 2.48^##^	2.43 ± 0.57^**^	1.52 ± 0.24^**^	2.74 ± 0.83^**^
Week 10	1.11 ± 0.24	9.36 ± 3.96^##^	4.26 ± 1.44^**^	2.97 ± 1.41^**^	3.70 ± 1.14^**^
TG (mmol/L)					
Week 6	0.58 ± 0.16	1.80 ± 0.47^##^	1.09 ± 0.32^**^	0.85 ± 0.10^**^	0.88 ± 0.27^**^
Week 10	0.62 ± 0.1	2.26 ± 0.82^##^	0.88 ± 0.14^**^	1.39 ± 0.63^*^	1.30 ± 0.42^*^
HDL-C (mmol/L)					
Week 6	0.65 ± 0.09	1.92 ± 0.57^##^	1.04 ± 0.24^**^	0.83 ± 0.16^**^	1.04 ± 0.21^**^
Week 10	0.68 ± 0.14	2.51 ± 0.74^##^	1.76 ± 0.67^*^	1.29 ± 0.57^**^	1.64 ± 0.37^*^
LDL-C (mmol/L)					
Week 6	0.44 ± 0.13	3.21 ± 1.85^##^	1.07 ± 0.12^**^	0.62 ± 0.20^**^	1.24 ± 0.48^*^
Week 10	0.46 ± 0.13	3.79 ± 2.20^##^	1.76 ± 0.50^**^	1.07 ± 0.61^**^	1.69 ± 0.86^*^

Control: rabbits fed on normal diets; Model: rabbits fed on high-cholesterol diets; Fenofibrate: rabbits received high-cholesterol diets plus fenofibrate (12 mg/kg); Kaempferol-H: rabbits received high-cholesterol diets plus kaempferol (150 mg/kg); Kaempferol-L: rabbits received high-cholesterol diets plus kaempferol (30 mg/kg). TC: total cholesterol; TG: triglyceride; HDL-C: high density lipoprotein cholesterol; LDL-C: low density lipoprotein cholesterol. ^*^P<0.05, ^**^p < 0.01, compared with model group; ^##^ p < 0.01, compared with control group.

### Serum inflammatory factors analysis

As has mentioned in Background, TNF-α and IL-1β are two important inflammatory factors in the progression of atherosclerosis. To evaluate the effect of kaempferol on the inflammation of vascular, we detected the serum levels of these two inflammatory factors in the present study. As listed in Table 3, serum TNF-α and IL-1β levels in rabbits of model group, which received high-cholesterol diet for ten weeks, increased notably as compared with the control group (P<0.01). However, in contrast to the model group, the concentrations of TNF-α and IL-1β in the group receiving high cholesterol diet plus fenofibrate (12 mg/kg) or kaempferol (30 mg/kg and 150 mg/kg) decreased significantly (P<0.05), indicating that kaempferol has an effect on TNF-α and IL-1β releasing.

**Table 3:** Changes of inflammatory factors in serum (mean± SD, n=6)

**Group**	**TNF-α (pg/mL)**	**IL-1β (pg/mL)**
Control	59.18 ± 11.23	14.46 ± 1.91
Model	143.93 ± 45.64^##^	27.66 ± 8.11^#^
Fenofibrate	92.82 ± 24.21^*^	17.16 ± 7.38^*^
Kaempferol-H	80.84 ± 29.69^*^	19.29 ± 3.12^*^
Kaempferol-L	83.76 ± 21.34^*^	20.02 ± 5.25^*^

Control: rabbits fed on normal diets; Model: rabbits fed on high-cholesterol diets; Fenofibrate: rabbits received high-cholesterol diets plus fenofibrate (12 mg/kg); Kaempferol-H: rabbits received high-cholesterol diets plus kaempferol(150 mg/kg); Kaempferol-L: rabbits received high-cholesterol diets plus kaempferol (30 mg/kg). TNF-α: Tumour-necrosis factor-α, IL-1β: interleukin-1β.^*^P<0.05, compared with model group; ^#^ p < 0.05, ^##^ p < 0.01, compared with control group.

### Serum antioxidation analysis

To evaluate the antioxidant effect of kaempferol on vascular in our experiment, we detected the serum levels of SOD and MDA, which are two commonly used standards to access antioxidant ability. The results were listed in Table 4. We can drawn from the table that, comparing to the control group, serum SOD levels of rabbits in model group dropped greatly (P<0.01), while serum MDA levels increased remarkably after high cholesterol diet for six and ten weeks. After ten weeks, levels of SOD increased and levels of MDA reduced significantly in the group treated with fenofibrate and kaempferol (P<0.05) compared with the model group. However at the sixth week, in rabbits treated with low dosage of kaempferol (30 mg/kg), the SOD and MDA levels were not significantly different from the model group (P>0.05). Six weeks may be too short for the drug kaempferol to exhibit a notable impact on the SOD and MDA levels.

**Table 4:** Changes of serum SOD and MDA after six and ten weeks in different groups (mean± SD, n=6)

	**SOD (μmol/L)**		**MDA (μmol/L)**	
**Group**	**Week 6**	**Week 10**	**Week 6**	**Week 10**
Control	119.00±15.01	106.76±33.56	1.86 ±0.69	2.26±0.70
Model	75.31±18.76^##^	32.64±11.31^##^	6.96 ±2.02^##^	9.29±3.15^##^
Fenofibrate	106.59±23.84^*^	85.68±23.32^**^	2.65±0.89^**^	5.89±0.92^*^
Kaempferol-H	101.32±24.60^*^	76.39±23.17^**^	5.10±1.38^*^	6.19±1.07^*^
Kaempferol-L	96.73±23.99	59.84±32.56^*^	5.49±1.77	6.43±1.22^*^

Control: rabbits fed on normal diets; Model: rabbits fed on high-cholesterol diets; Fenofibrate: rabbits received high-cholesterol diets plus fenofibrate (12 mg/kg); Kaempferol-H: rabbits received high-cholesterol diets plus kaempferol(150 mg/kg); Kaempferol-L: rabbits received high-cholesterol diets plus kaempferol (30 mg/kg). MDA: malondialdehyde; SOD: superoxide dismutase.^*^P<0.05, ^**^p<0.01, compared with model group; ^##^p < 0.01, compared with control group.

### Morphologic and histologic studies

**Figure 2:**
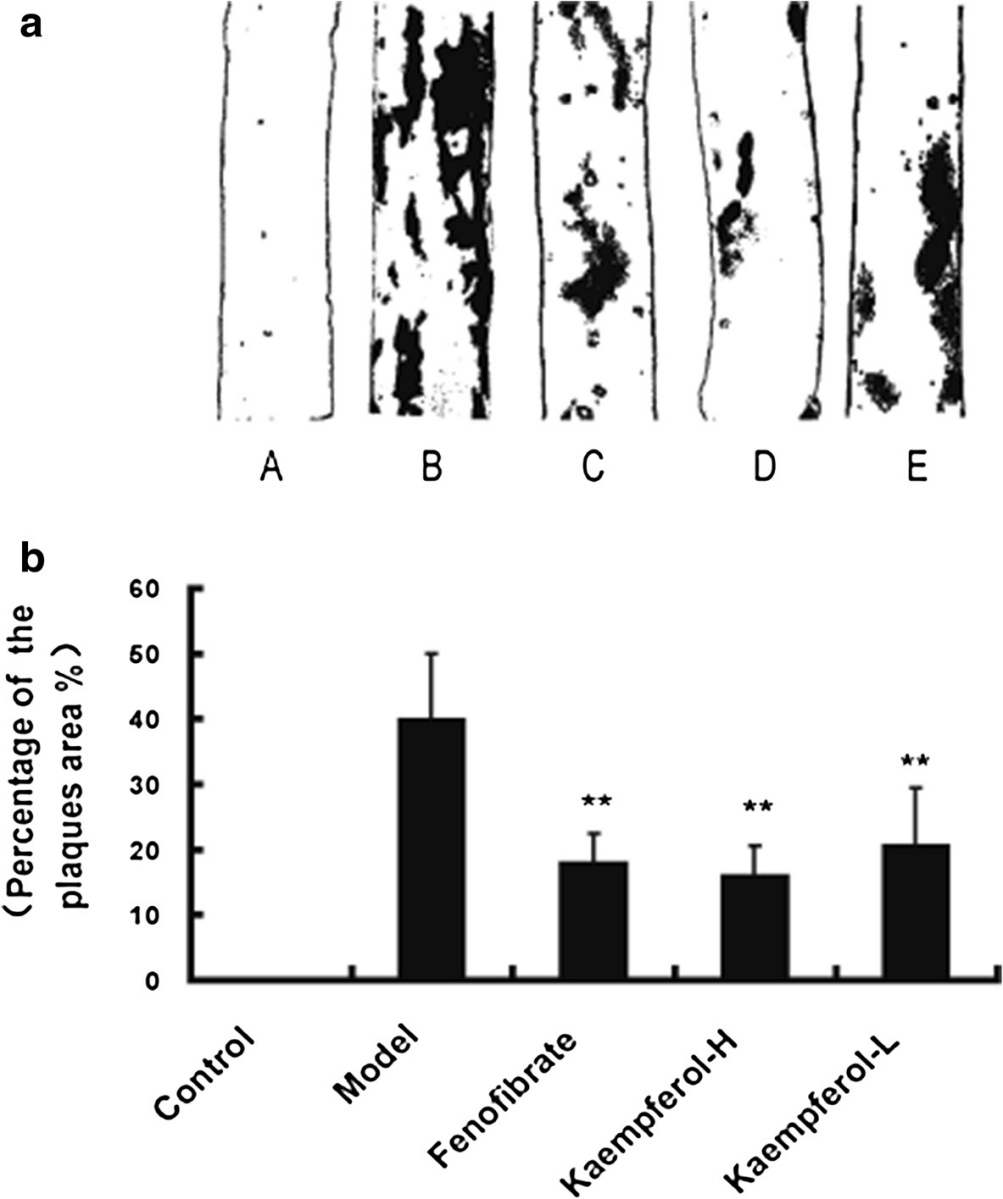
**Effect of kaempferol on plaque and fatty steak formation in the aorta of rabbits. (a)** Representative photographs of oil red O stained aorta of the third portion; **(b)** Percentage of the plaques area on the third portion aorta of rabbits in each group. Expressed as percentage of the oil red-O positive area/total internal surface area. **A**. Control; **B**. Model; **C**. Fenofibrate; **D**. Keampferol-H; **E**. Keampferol-L. Each bar represents mean+SD (n=6). ^**^P <0.01 compared with control group.

After oil red O staining of the third aorta (between the third and seventh intercostals artery), atherosclerosis lesions on the aorta were stained red. Examples of obtained aorta stained by red oil in each groups are shown in Figure 2a. Grossly, aortas of rabbits of the high-cholesterol-fed model group induced deposition of lipids and thickening of the intima. Small plaques, 0.5 to 5 mm diameter, and wide areas of fatty streak lesions can be seen in the aortas of model group, while the appearance of aortic internal surface in control group was normal and no plaques or fatty streak had been observed. The fenofibrate-supplemented and kaempferol-supplemented groups developed only small and thinner plaques at the obtained aorta portion. Percentage of the plaques area was expressed as the percentage of the aorta stained positively for oil red O on third portion and was calculated with computer software. Comparing to the control group, the percentage of the plaques area changed largely in the aorta of model rabbits (p<0.01). However, it significantly decreased in fenofibrate group (17.79±4.69%), kaempferol-H group (16.01±4.58%) and kaempferol-L group (20.52±8.93%) as compared with the model group (39.79±10.20%) (P<0.01, Figure 2b).

**Figure 3:**
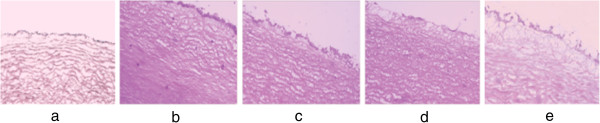
**Cross-section of the first portion of aorta stained with hematoxylin and eosin (H&E) under light microscope (magnification×100). a**. Control; **b**. Model; **c**. Fenofibrate; **d**. Keampferol-H; **e**. Keampferol-L.

Representative images of the first portion of aorta (one centimeter segment between the first and the second intercostals artery) stained by H&E under light microscope were shown in Figure 3. As we can see, the aortic segment from control group was normal and integrity, the intima was composed of a thin endothelial cell layer and elastic fibers layer and smooth muscle cells (SMCs). While the aorta from rabbits fed on a high-cholesterol diet exhibited obvious signs of AS, foam cells and cholesterol deposits were largely found in the intima. The luminal endothelium was damaged and the medial smooth muscle cells underwent proliferation. Samples of those in groups treated with fenofibrate (12 mg/kg) and keampferol (30 mg/kg, 150 mg/kg), showed less accumulation of lipids and less damage of intima. Thinner intima was observed compared to model group, though there were still a few macrophages and scattered foam cells, especially in group treated with kaempferol.

### Immunohistochemical findings

**Figure 4:**
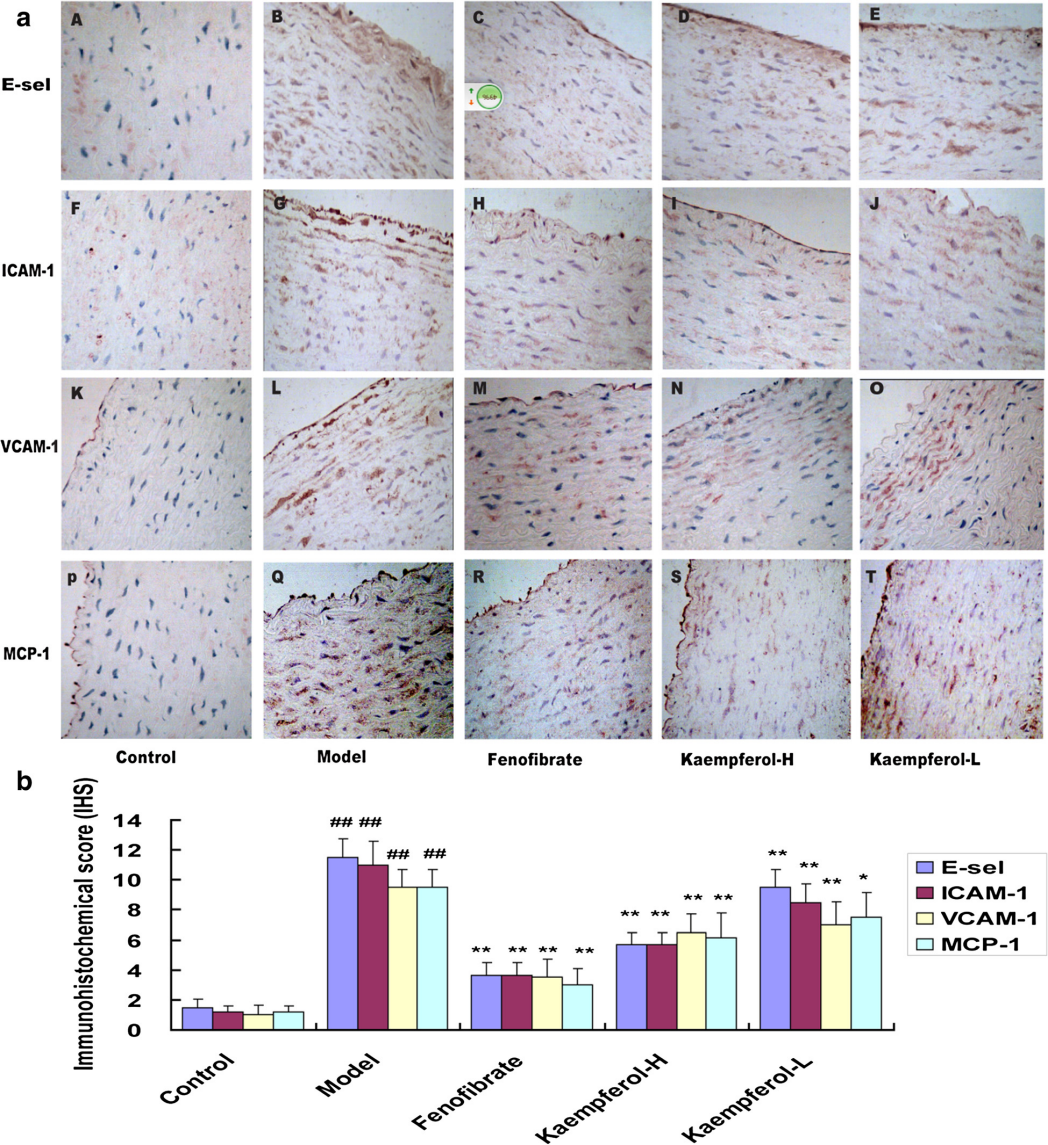
**Results of immunohistochemical study. a**. Representative examples of immunohistochemical staining of E-sel (A-E), ICAM-1 (F-J), VCAM-1 (K-O) and MCP-1 (P-T) in the first portion of rabbit aortas (amplification ×400). **b**. Immunohistochemical score (IHS) of rabbit aorta sections. Control: rabbits fed on normal diets; Model: rabbits fed on high-cholesterol diets; Fenofibrate: rabbits received high-cholesterol diets plus fenofibrate (12 mg/kg); Kaempferol-H: rabbits received high-cholesterol diets plus kaempferol (150 mg/kg); Kaempferol-L: rabbits received high-cholesterol diets plus kaempferol (30 mg/kg). Each bar represents mean± SD (n = 6). ^*^P<0.05, ^**^p < 0.01, compared with model group; ^##^ p < 0.01, compared with control group.

E-sel, ICAM-1, VCAM-1and MCP-1 expression of the first portion of the aorta obtained at the end of tenth week (one centimeter segment between the first and the second intercostals artery) was analyzed immunohistochemically. Respective images of immunstains obtained from each animal in every study group were shown in Figure 4a, and the immunohistochemical score (IHS) of E-sel, ICAM-1, VCAM-1 and MCP-1 expression were pictured in Figure 4b. As we can see, sections of control group did not show any staining (Figure 4a A, F, K and P). On the contrary, E-sel, ICAM-1, VCAM-1 and MCP-1 were strongly expressed in model rabbits fed with high-cholesterol diet (Figure 4a B, G, L and Q). Fenofibrate group, Kaempferol-H and Kaempferol-L group all showed weak expression of E-sel, ICAM-1, VCAM-1 and MCP-1(Figure 4a C-E, H-J, M-O and R-T). According to Figure 4b, the scores of E-sel, ICAM-1, VCAM-1 and MCP-1 expression in normal rabbits were much low (1.50±0.55 for E-sel, 1.17±0.41 for ICAM-1, 1.00±0.63 for VCAM-1, 1.17±0.41 for MCP-1), however, they raised up greatly to a high level (11.50±1.22, 11.00±1.55, 9.50±1.22 and 9.50±1.22 respectively) in model group with the inducing of high-cholesterol diet. In positive control rabbits (supplemented with fenofibrate), the expressions of E-sel, ICAM-1 and VCAM-1 and MCP-1 were markedly reduced compared with model group (P<0.01). 10 weeks of high-cholesterol diet supplemented with kaempferol (30 mg/kg and 150 mg/kg) significantly lowed scores of E-sel, ICAM-1, VCAM-1 and MCP-1 expression on cells in the aorta compared to model rabbits (P<0.01). However, down-regulation of VCAM-1 and MCP-1 expression on cells of the aorta of rabbits with kaempferol supplemented diet, did not reach a statistical significance between the high dosage group and the low dosage group (P > 0.05).

### Gene expression of E-sel, ICAM-1, VCAM-1 and MCP-1

**Figure 5:**
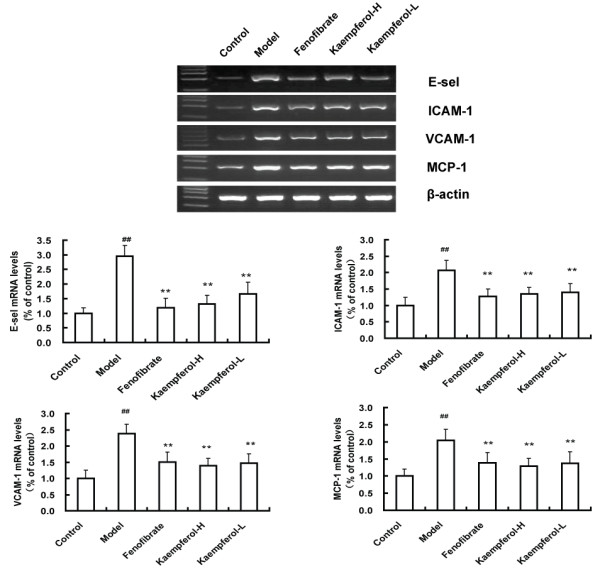
**Results of gene expression by Semi-quantitative RT-PCR.** Representative gel images of E-sel, ICAM-1, VCAM-1 and MCP- 1 mRNA expression of the second portion of rabbit aortas. β-actin was used as an internal control and PCR products were visualized by ethidium bromide staining. The data in the bar graph are quantified ratios of the signal for E-sel, ICAM-1, VCAM-1 and MCP-1 to that for β-actin with the control samples set at 100%. Results are expressed as mean± SD (n = 6). ^**^ p <0.01 compared with the model group, ^##^ p < 0.01, compared with control group.

mRNA expression of E-sel, VCAM-1, ICAM-1 and MCP-1 at the second portion aorta (one centimeter segment between the second and the third intercostals artery) were studied in each individual arteries by a semi-quantitative PCR as described in Materials and methods. Results were expressed as ratios between E-sel, ICAM-1,VCAM-1 or MCP-1 and β-actin. Representative gel images of PCR obtained from each animal in every study group were shown in Figure 5. As presented in Figure 5, samples in control animals showed normal mRNA expression of E-sel, VCAM-1, ICAM-1 and MCP-1, while significantly increased expression of these genes in model animals was noticed (P<0.05). In addition, the mRNA expression of E-sel, ICAM-1, VCAM-1 and MCP-1 were significantly lower in the fenofibrate and kaempferol treated animals than in the model group (P <0.01), especially in high dosage kaempferol treated group. However, the differences between kaempferol-H and kaempferol-L groups were not significant (P>0.05).

## Discussion

According to several epidemiological and experimental studies, natural or synthetic products having an anti-inflammatory action have been proven to have a strong preventive effect on the development of atherosclerosis [19,20]. To investigate the potential anti-inflammatory activity of kaempferol as a new anti-atherogenic agent, the present study was designed to evaluate the role of kaempferol on the inflammatory progression of early atherosclerosis in rabbits. After ten weeks of kaempferol treatment together with high-cholesterol diet, blood lipids and arterial lesions of rabbits were significantly deduced compared to the untreated group. Levels of serum IL-1β and TNF-a dropped, mRNA and protein expression of E-sel, VCAM-1, ICAM-1 and MCP-1 in the aorta of rabbits were respectively down-regulated by the treatment of kaempferol, indicating that kaempferol can prevent atherosclerosis by alleviating vascular inflammation.

Selectin belongs to one kinds of adhesion molecules.Selectin family is divided into three categories: E-selectin, P-selectin and L-selectin. They play an important role in vascular inflammation, thrombus and plaques formation, atherosclerosis, vascular vasodilation, metabolism of vascular endothelial cells. E-selectin (E-sel) is a cytokine select selectin and mainly located in the capillaries and endothelial cell membrane of venules. Expression of E-sel on non-activated endothelial cells is minimal, but it rise up greatly when stimulated by inflammatory cytokines like IL-1β, TNF-α and lipopolysaccharide, and this change is induced at the transcriptional level [5]. ICAM-1 and VCAM-1 are two frequently studied adhesion molecules that are cytokine-inducible members of immunoglobulin gene superfamily, they are highly expressed by arterial endothelial cells at the atherosclerotic lesions [21]. It is well established that E-sel, VCAM-1, ICAM-1 and MCP-1are key molecules involved in the initiation of inflammation pathogenesis of atherosclerosis, they collaboratively promote the adhesion of monocytes and then the migration to endothelium. Previous reports found that the expression of VCAM-1 and ICAM-1are up-regulated by a high cholesterol diet [22,23]. The high expression of E-sel, ICAM-1 and VCAM-1 were also detected mainly in human atherosclerotic plaques [7]. Levels of soluble E-sel, ICAM-1 and VCAM-1 in blood plasma were significantly higher in patients with unstable angina, acute coronary syndrome and atherosclerosis. These adhesion molecules are becoming potent clinical biomarkers of CVD [24]. Monocyte chemoattractant protein-1 (MCP-1) is thought to play a central role in filtration of monocyte /macrophage to the arterial wall and the recruitment of these cells [25]. It has been demonstrated that MCP-1 expression occurs in the arterial wall in response to hypercholesterolemia in rabbits and MCP-1 is highly expressed in the macrophage -rich area of the atherosclerotic lesions in human and animal models [26]. Increases in MCP-1 levels in serum have also been observed in patients with acute coronary syndromes and expression of vascular MCP-1 can be used to predict atherosclerosis [27]. Also, it was revealed that TNF-α, IL-1β as well as oxidized low density lipoprotein (LDL) were able to induce local vascular cells to produce MCP-1, which causes monocyte recruitment and promotes the release of lipids, thereby enhancing the progression of the atherosclerotic lesion [28,29]. Another report found that the inhibition of MCP-1 expression by gene targeting could reduce atherogenesis in LDL receptor deficient mice [30].

According to some studies, flavonoids including phloretin [31], apigenin [32] and quercetin [33,34] can inhibit E-sel, ICAM-1, VCAM-1 or MCP-1 expression in vitro and in vivo. In our study, mRNA and protein expressions of E-sel, ICAM-1, VCAM-1 and MCP-1 in aorta of rabbits were all up-regulated by a high-cholesterol diet and expressions of those three molecules were all down-regulated by kaempferol (30 mg/kg and 150 mg/kg). Therefore, similar to the other flavonoids mentioned above, kaempferol also showed a down-regulation effect on E-sel, ICAM-1, VCAM-1 or MCP-1 expression in vivo.

On the initiation of inflammation, inflammatory stimuli leads to increased secretion of pro-inflammatory cytokines such as TNF-α and IL-1β, which can significantly induce oxidation of LDL and promote aggregation of oxidized LDL and macrophages, thereby lead to the formation of foam cells, increase the binding of LDL on endothelial cells and the expression of LDL receptor of endothelial cells. Subsequently nuclear transcription factor-kappa B (NF-κB) pathway is activated in response to pro-inflammatory cytokines. This pathway plays an essential role in inflammation through the regulation of genes encoding pro-inflammatory cytokines, adhesion molecules, chemokines and growth factors. The activation of these genes will stimulating the migration and proliferation of smooth muscle cells, promoting the development of atherosclerotic plaques [35]. Studies demonstrated the activation of gene expression of endothelial cell adhesion molecules including E-sel, ICAM-1 and VCAM-1 by IL-1β or TNF-α in endothelial cells is mediated by NF-κB pathway [36]. Increased IL-1β protein [37] as well as TNF-α mRNA and protein expression [38] are found in injured arterial smooth muscle cells, and the inhibition of TNF-α treatment can accelerate endothelial recovery after injury [39]. Besides, the disruption of TNF-α gene diminishes the development of atherosclerosis in ApoE-deficient mice [40]. A resent study demonstrated that an antibody targeting IL-1β can inhibit the progression of atherosclerosis in vivo, highlighting the importance of this key cytokine in cardiovascular disease [41].Therefore, IL-1β and TNF-α are two important pro-inflammatory cytokines in the initiation and development of atherosclerosis.

Several studies on the anti-inflammation effect of kaempferol in vitro have also shown that kaempferol can down-regulate TNF-α and IL-1β expression [42,43]. In our study, which centering on the in vivo anti-inflammatory activity of kaempferol, we demonstrated that kaempferol can inhibit inflammatory cell adhesion, migration and block the initiating processes of inflammatory response. The effect may be medicated by reducing the secretion and release of inflammatory mediators such as TNF-α and IL-1β and decreasing the expression of the E-sel, ICAM-1, VCAM-1 as well as MCP-1 in the aorta of rabbits. And both high and low dosage of kaempferol (150 mg/kg and 30 mg/kg) have a strong effect both on the gene and protein expression, but the effect was not dosage related, for there were no statistic difference between the two groups. Maybe the treatment dosages designed in our study were not in the linear efficacy range of kaempferol. In addition, the serum TNF-α and IL-1β levels were increased in atherosclerosis rabbits, and kaempferol groups showed lower TNF-α and IL-1β levels, indicating that the down-regulation of E-sel, ICAM-1, VCAM-1 and MCP-1 might be mediated by the reducing of TNF-α and IL-1β.

Besides, our results suggested that kaempferol have anti-oxidant effect on high cholesterol fed rabbits, for that increased SOD and reduced MDA were observed six and ten weeks post kaempferol administration. It has been known that free radicals is highly related with caediovascular disease and antioxidants including many natural agents can protect the developing of CVD [44,45]. According to some studies, kaempferol is a potent and powerful superoxide scavenger [46]. The ability of kaempferol to decrease superoxide levels by inhibiting the activity of enzymes that generate reactive oxygen species (ROS), such as the enzyme xanthine oxidase, may play a crucial role in its antioxidant activity [47]. Many reports have shown that kaempferol and several kaempferol-containing plants have antioxidant activity not only in vitro, but also in vivo [48,49]. Several epidemiological studies reported that fruit and vegetables rich in kaempferol can prevent cardiovascular diseases, and intake of them reduces the risk of coronary heart disease. The antioxidant effect of kaempferol observed in our experiment may be mediated by protecting LDL from oxidation, which is also one of the early processes involved in atherosclerosis [50].

## Conclusion

In summary, the present study suggested that six and ten weeks of treatment of kaempferol (30 mg/kg, 150 mg/kg) effectively prevent atherosclerosis induced by high cholesterol in New Zealand White rabbits. The anti-atherogenic effect possibly works by improving antioxidant ability, reducing the release of TNF-α and IL-1β and down-regulation the gene and protein expression of pro-atherogenic molecules, such as E-sel, ICAM-1, VCAM-1 and MCP-1.Further studies related to the anti-inflammatory actions of keampferol may needed to provide more evidences for its anti-atherosclerotic effect.

## Abbreviations

TNF-α: Tumour-necrosis factor-alpha; IL-1β: Serum interleukin-1beta; MDA: Malonaldehyde; SOD: Superoxide dismutase; E-sel: E-selectin; ICAM-1: Intercellular adhesion molecule-1; VCAM-1: Vascular cell adhesionmolecule-1; MCP-1: Monocyte chemotactic protein-1; CVD: Cardiovascular disease; RT-PCR: Reverse transcription-polymerase chain reaction; LDL: Low density lipoprotein; TC: Total cholesterol; TG: Triglyceride; HDL-C: High density lipoprotein cholesterol; LDL-C: Low density lipoprotein cholesterol.

## Competing interests

The authors declare that they have no competing interests.

## Authors’ contributions

LK carried out most of the experiment and involved in manuscript drafting; CL performed part of the experiment and participated in editing and revising of the manuscript; XL was involved in statistical analysis and revising the manuscript critically for important intellectual content. YZ was involved in project supervision and study design. HH was responsible for part of the experiment implement and manuscript revising. All authors have read and approved the final manuscript.
